# Draft genome sequence of a family Acutalibacteraceae isolate, a human gut-derived cholesterol metabolizer

**DOI:** 10.1128/mra.01137-25

**Published:** 2026-03-10

**Authors:** Stephen Skolnick, Brantley Hall

**Affiliations:** 1 Constellation Bio, Berkeley, California, USA; 2 Department of Cell Biology and Molecular Genetics, University of Maryland 1068 , College Park, Maryland, USA; 3 Center for Bioinformatics and Computational Biology, University of Maryland 1068 , College Park, Maryland, USA; Wellesley College, Wellesley, Massachusetts, USA

**Keywords:** human gut microbiome, cholesterol, coprostanol

## Abstract

We report the draft genome sequence of a family Acutalibacteraceae isolate, which is an obligate anaerobe that converts cholesterol to coprostanol. The organism was isolated from a healthy adult’s fecal sample, plated on cholesterol brain agar, and incubated anaerobically, yielding distinctive star-shaped colonies. This isolate is from an unclassified genus within the Acutalibacteraceae, highlighting the unexplored diversity within the gut microbiome.

## ANNOUNCEMENT

There is increasing evidence that human gut microbes not only metabolize cholesterol but also have a significant impact on human serum cholesterol (1)*.* Despite this, only a few cholesterol-metabolizing microbes have been isolated, leaving significant barriers to understanding microbial sterol metabolism and its impact on human health. To understand the taxa and mechanisms involved in these pathways, effort needs to be put toward isolating and characterizing novel cholesterol-metabolizing strains.

Cholesterol brain agar (CBA) was prepared as described by Brinkley, Gottesman, and Mott (2) and pre-reduced overnight in an anaerobic chamber (5% CO_2_, 5% H_2_, and 90% N_2_). A fresh fecal sample from a healthy 32-year-old man with favorable serum lipids (low-density lipoprotein cholesterol [LDL-C] 90 mg/dL and high-density lipoprotein cholesterol [HDL-C] 89 mg/dL) was transferred to the chamber within 1 min of excretion. After homogenization and serial dilution in liquid brain medium, the cultures were plated onto CBA and incubated anaerobically at 35°C for 7 days.

Well-isolated colonies displaying a characteristic “fibrous” morphology, consisting of a central colony with thin arm-like extensions spreading out in all directions, were identified. One colony was picked, restreaked for purity, and inoculated into liquid cholesterol-brain medium. The culture exhibited “clotting” behavior, forming clumps in the media. This provided direct evidence of bacterial cholesterol conversion to coprostanol and resulted in crystalline fiber precipitation that solidified the medium. This behavior was absent in the sterile control medium. Stocks were prepared by combining solidified medium with 40% glycerol (1:1 ratio).

**Fig 1:**
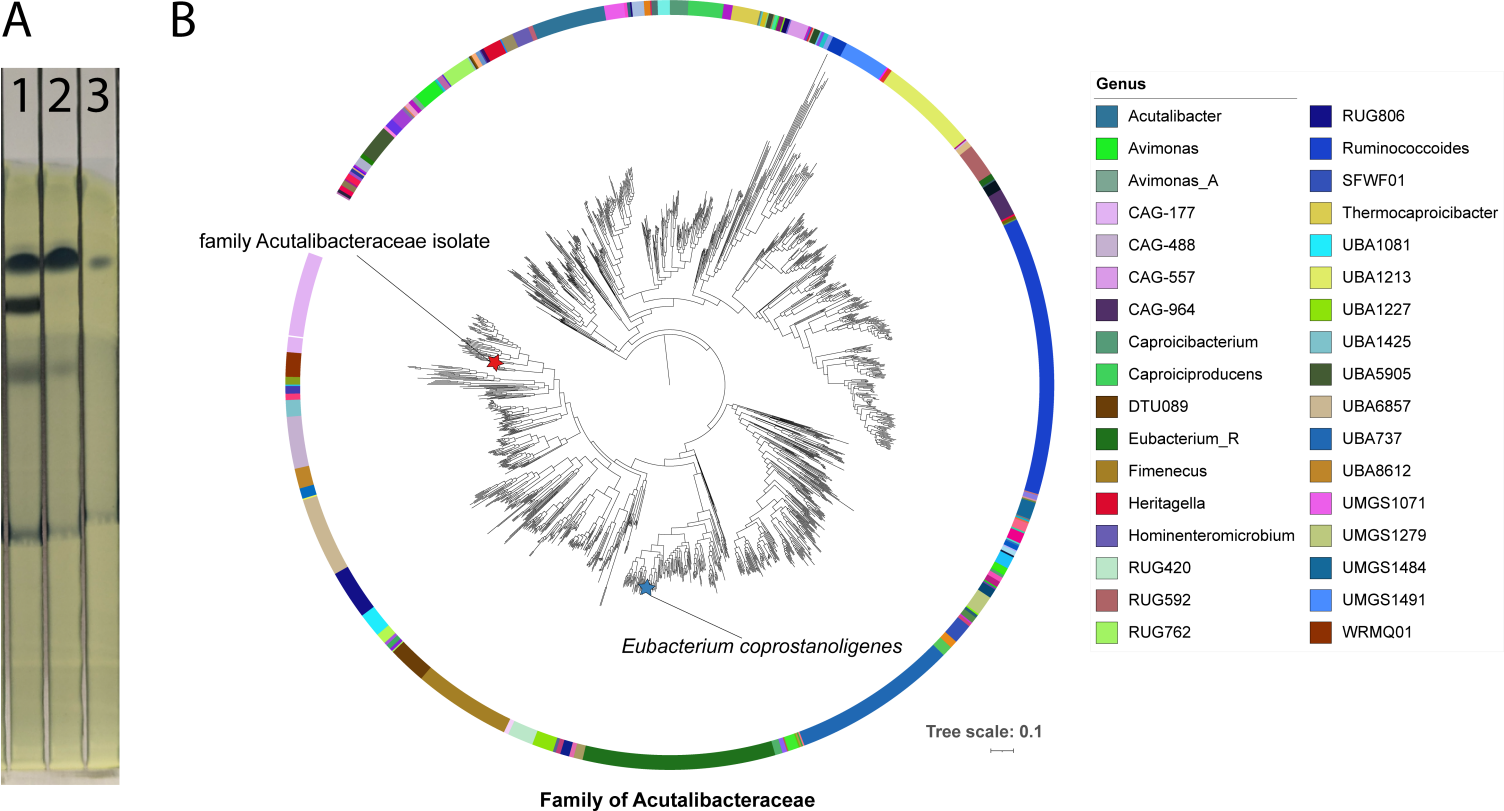
(**A**) Validation of coprostanol production by thin-layer chromatography. All lanes: hexane extract (10 µL/mg unless otherwise stated); 20 µL spotted. Lane 1: Cholesterol standard (Carl Roth), 125 µg/mL. Lane 2: CBM, sterile. Lane 3: CBM after fermentation by the family Acutalibacteraceae isolate. (**B**) Phylogenetic placement demonstrating that the family Acutalibacteraceae isolate represents a distinct evolutionary lineage within the Firmicutes phylum.

To test for coprostanol production by thin-layer chromatography, silica gel plates were pre-treated with 5% phosphomolybdic acid in ethanol. Samples (sterile and fermented medium) were hexane-extracted for ≥1 h at room temperature, and 20 µL aliquots were spotted onto plates (3). Running solvent (diethyl ether:heptane, 55:45) separated cholesterol (Rf 0.3) from coprostanol (Rf 0.4). Coprostanol appeared consistently in fermented medium, but not in sterile controls (Fig. 1A)*.*

Using samples of the strain culture, genomic DNA extraction and library preparation were performed by AmpSeq (Gaithersburg, MD) using ZymoBIOMICS (Zymo Research) and NEBNext Ultra II (New England Biolabs) kits as per the manufacturer’s recommendations. Sequencing on the Element AVITI platform (2 × 150 bp) generated 14,173,132 high-quality paired-end reads (>Q30), of which 10,698,020 remained after quality trimming with Trimmomatic (4) to remove TruSeq3 adapters, remove the leading and lagging three base pairs, and only keep reads with a minimum length of 36 base pairs. *De novo* assembly with SPAdes (5) v4.2.0 with default parameters yielded seven contigs totaling 2,394,193 bp, following the removal of contigs smaller than 1 kb. The final assembly was with 200× coverage, 50.97% GC content, and N50 of 1,711,562 bp. Prokka (6) v1.14.6 annotation using default settings identified 2,220 protein-coding sequences (Table 1).

**TABLE 1:** Assembly statistics for the Acutalibacteraceae isolate

Assembly statistic	Value
Genome size (bp)	2,394,193
N50 contig size (kb)	1,711,562
Number of contigs	*7*
Largest contig	1,711,562
Total reads	14,173,132
GC (%)	50.97
Number of protein-coding genes	2,200

Analysis with GTDB-Tk v2.4.0 (release 226) (7) revealed that the family Acutalibacteraceae isolate represents a distinct evolutionary lineage within Firmicutes, phylogenetically distant from *Eubacterium coprostanoligenes* ATCC 51222, the previously characterized cholesterol-reducing bacterium (1, 8). The two species occupy separate clades within a broader group containing 567 genomes (Fig. 1B). Based on the phylogenetic reconstruction, the closest related genome to the family Acutalibacteraceae isolate is CAG-177 sp003514385 (GCA_003514385.1), a metagenome-assembled genome (MAG) derived from a healthy human gut sample (9). The tree was generated with GTDB-Tk’s de novo workflow using bac120 markers and FastTree (10*)* v 2.2.

Only *E. coprostanoligenes* and this family Acutalibacteraceae isolate are cultured isolates among these 567 genomes, and the remaining 565 are MAGs. This underscores severe cultivation challenges within this group and highlights this isolate’s significance as only the second cultured and sequenced representative of this extensive clade of cholesterol-metabolizing bacteria.

## Data Availability

This Whole Genome Shotgun project has been deposited in GenBank under the accession no. JBPPLN000000000. Illumina reads have been deposited to the NCBI SRA under accession number SRR35768769.

## References

[1] Kenny DJ, Plichta DR, Shungin D, Koppel N, Hall AB, Fu B, Vasan RS, Shaw SY, Vlamakis H, Balskus EP, Xavier RJ. 2020. Cholesterol metabolism by uncultured human gut bacteria influences host cholesterol level. Cell Host Microbe 28:245–257. doi:10.1016/j.chom.2020.05.013 32544460 PMC7435688

[2] Brinkley AW, Gottesman AR, Mott GE. 1982. Isolation and characterization of new strains of cholesterol-reducing bacteria from baboons. Appl Environ Microbiol 43:86–89. doi:10.1128/aem.43.1.86-89.1982 6798934 PMC241785

[3] Hoskin GP, Bandler R. 1987. Identification of mammalian feces by coprostanol thin layer chromatography: method development. J Assoc Off Anal Chem 70:496–498. 3112114

[4] Bolger AM, Lohse M, Usadel B. 2014. Trimmomatic: a flexible trimmer for Illumina sequence data. Bioinformatics 30:2114–2120. doi:10.1093/bioinformatics/btu170 24695404 PMC4103590

[5] Bankevich A, Nurk S, Antipov D, Gurevich AA, Dvorkin M, Kulikov AS, Lesin VM, Nikolenko SI, Pham S, Prjibelski AD, Pyshkin AV, Sirotkin AV, Vyahhi N, Tesler G, Alekseyev MA, Pevzner PA. 2012. SPAdes: a new genome assembly algorithm and its applications to single-cell sequencing. J Comput Biol 19:455–477. doi:10.1089/cmb.2012.0021 22506599 PMC3342519

[6] Seemann T. 2014. Prokka: rapid prokaryotic genome annotation. Bioinformatics 30:2068–2069. doi:10.1093/bioinformatics/btu153 24642063

[7] Chaumeil P-A, Mussig AJ, Hugenholtz P, Parks DH. 2022. GTDB-Tk v2: memory friendly classification with the genome taxonomy database. Bioinformatics 38:5315–5316. doi:10.1093/bioinformatics/btac672 36218463 PMC9710552

[8] Ren D, Li L, Schwabacher AW, Young JW, Beitz DC. 1996. Mechanism of cholesterol reduction to coprostanol by Eubacterium coprostanoligenes ATCC 51222. Steroids 61:33–40. doi:10.1016/0039-128x(95)00173-n 8789734

[9] Bengtsson-Palme J, Angelin M, Huss M, Kjellqvist S, Kristiansson E, Palmgren H, Larsson DGJ, Johansson A. 2015. The human gut microbiome as a transporter of antibiotic resistance genes between continents. Antimicrob Agents Chemother 59:6551–6560. doi:10.1128/AAC.00933-15 26259788 PMC4576037

[10] Price MN, Dehal PS, Arkin AP. 2009. FastTree: computing large minimum evolution trees with profiles instead of a distance matrix. Mol Biol Evol 26:1641–1650. doi:10.1093/molbev/msp077 19377059 PMC2693737

